# Validating the use of clinical MSCT scans for cranial nonmetric sex estimation in a contemporary Indonesian population

**DOI:** 10.1007/s00414-024-03176-5

**Published:** 2024-02-01

**Authors:** Ridhwan Lye, Zuzana Obertová, Nur Amelia Bachtiar, Daniel Franklin

**Affiliations:** 1 Centre for Forensic Anthropology, M420, The University of Western Australia, Crawley, WA 6009 Australia; 2 Radiology Department, Hasanuddin University, Jalan Perintis Kemerdekaan KM. 10, Talamanrea, Makassar, 90254 Indonesia

**Keywords:** Forensic anthropology, Sexual dimorphism, Sex estimation, Cranial nonmetric traits, Logistic regression, Indonesia

## Abstract

There is renewed interest in Asia for the development of forensic anthropological standards, partly due to the need for population-specific models to maintain high classification accuracies. At present, there are no known studies utilising morphoscopic standards specific to the Indonesian population. Craniometric analyses can often be time-consuming; morphoscopic assessments are quicker and are also known to be reliable and accurate. One of the most utilised morphoscopic standards for the estimation of skeletal sex is that of Walker (2008). Its application across population groups demonstrated reduced accuracies outside of the United States; population-specific predictive models would thus serve to improve the identification process of unknown skeletal remains. Digital imaging also allows for the validation of standards on a contemporary population and is an appropriate proxy to physical skeletal material.

The present study quantifies the applicability of the Walker standard to a contemporary Indonesian population. A total of 200 cranial MSCT scans from a hospital in Makassar were analysed using *OsiriX®*. Scoring was performed in accordance with the Walker standard. Five univariate and nine multivariate predictive models were derived using single trait and multi-trait combinations. The best performing univariate model included the glabella, with a total classification accuracy of 82.0% and a sex bias of 14.6%. Classification accuracy with all traits considered was at 95.2% for females and 82.8% for males with a sex bias of 12.5%. These results provide forensic practitioners in Indonesia with an appropriate morphoscopic sex estimation standard, strengthening their capabilities in the field and improving judicial outcomes.

## Introduction

The estimation of skeletal sex requires an understanding of the differences in skeletal architecture relative to distinct sex-specific biological processes and evolutionary theory, including musculoskeletal loading [1], pubertal growth trajectories, and sexual selection [2, 3]. In modern forensic practice, the skull is considered the second-most popular region for skeletal sex estimation [4]. Amongst the most frequently applied morphoscopic standard are the five cranial traits associated with the Walker [5] standard. Although originally published in *Standards for Data Collection from Human Skeletal Remains* [6], its subsequent republication expanded on the scoring-based system by introducing quantifiable measures of accuracy though discriminant functions derived from logistic regression [7–9].

The incorporation of quantifiable statistics into the estimation of skeletal sex in Walker [5] was an important step taken to address a major perceived disadvantage of morphoscopic standards, namely the reduced reliability and subjectivity when scoring traits based off visual assessments in comparison to using metric analysis [10, 11]. The improved statistical presentation of this morphoscopic approach has further strengthened its popularity among practitioners in both routine medicolegal casework and disaster victim identification (DVI). The preferential use of morphoscopic assessments has long been based on the ease of use of morphoscopic standards, which in comparison to metric analyses, do not require any specialised equipment and can be utilised with incomplete and fragmentary skeletal remains [4, 12].

Inter-population studies have assessed the applicability of the Walker [5] standard, with several reporting lower classification accuracies for females in a Greek population, at 53.57% [13], and also for females in an Italian population, at 78.6% [14], compared to 86.4% in Walker [5]. A South African study also reported reduced classification accuracies, with females classified below chance (i.e., < 50.0%) [15]. Japanese and Thai populations tested in Tallman [16] reported large misclassifications in both females and males, with accuracies as low as 26.3% and 30.2%, respectively, further emphasising the importance of utilising standards specific to their relevant population [12].

Clinical digital imaging, including computed tomography (CT) scans, has been validated for use with the Walker [5] standard. When tested on a Turkish population, Dereli et al. [17] reported classification accuracies between 83.3% and 86.1% for females and 92.9% and 100.0% for males by three observers. However, scorings for this study were done using *Standards* [6] without applying the regression functions in Walker [5]. When tested on a mixed US population group consisting of African, Asian, European, Latin, and Native Americans, Kelley and Tallman [18] reported classification accuracies between 84.2% and 92.1% for females and 77.1% and 93.2% for males, depending on the population-specific or pooled-population function derived from their study. These studies have allowed researchers to access more contemporary sampling as secular variation is known to affect the accuracy of skeletal sex estimates [19]. It also demonstrated the viability of virtual samples as a valuable alternative to physical skeletal collections.

Although there appears to be growing interest in the development of standards for use in Asia more broadly, these developments are limited by access to skeletal collections [20–22]. Cultural and religious restrictions related to invasive autopsies and the handling of deceased individuals must be taken into consideration [23, 24]. With the relatively high frequency of natural and human-induced mass fatality events in Indonesia [25, 26], standards that can be applied to fragmentary remains domestically are lacking.

The inclusion of CT scanning in the research design is two-fold. First, it adheres to and respects the cultural and religious significance of the deceased. Secondly, it allows forensic practitioners to scan and preserve physical skeletal material in a 3D environment, which can then be sent to other practitioners, especially in instances of DVI, significantly improving the speed and likelihood of positive identification [27].

As such, the aims of the present study are to evaluate the accuracy of skeletal sex estimation using the Walker [5] standard and other Asian-derived predictive models in Tallman [16], and to develop forensically applicable predictive models for the estimation of skeletal sex optimised for the contemporary Indonesian population.

## Materials and method

### Study sample

**Fig. 1:**
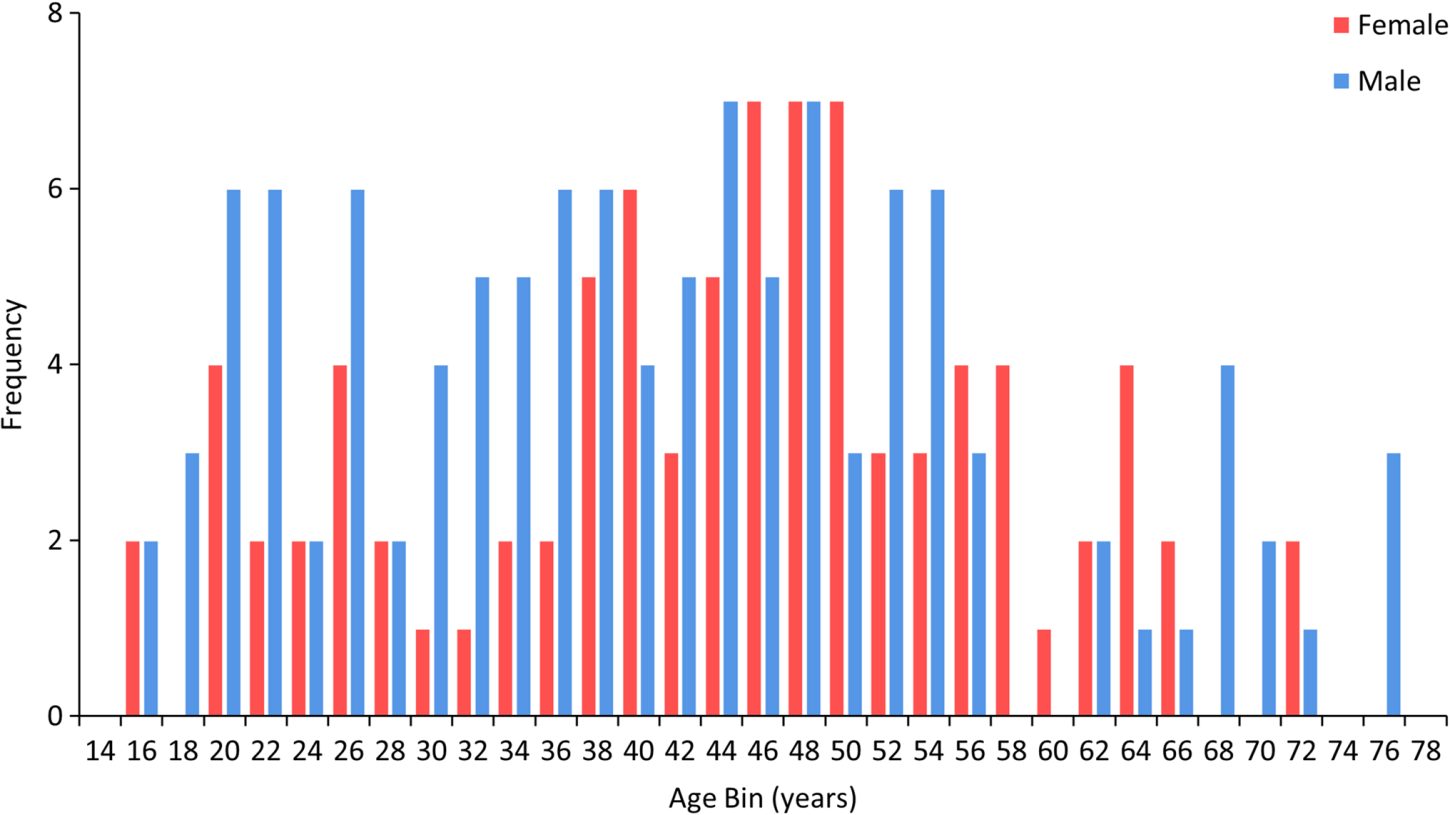
Frequency distribution of the Indonesian sample by sex and age

A total of 200 multi-slice CT scans (MSCT) were analysed for this study, comprising 87 female and 113 male individuals. Ages ranged from 15 to 76 years (female: mean age = 43.6 years, SD = 13.4 years; male: mean age = 40.8 years, SD = 15.2 years). Figure 1 shows the age and sex distribution of the sample.

The scans were obtained from the Picture Archiving and Communications System (PACS) database at the Dr Wahidin Sudiohusodo General Hospital (RSWS) within Hasanuddin University in Makassar, from patients who presented at the hospital for radiological examination as part of their normal course of treatment between February 2020 and August 2022. All CT scans were anonymised through PACS prior to receipt, except for recorded age and sex of individual patients at the time the scans were taken.

Imaging was performed with a Siemens Healthineers SOMATOM go.Top 128-slice CT scanner, with resolutions between 0.6 and 1.5 mm (58.0% of all scans are 1.0 mm). Scans that showed signs of acquired or congenital pathology, or other abnormalities that would obscure the observation of traits in the skull, were excluded from this study and therefore not included in the total sample count.

Approvals were provided by the Human Ethics division of the Office of Research at the University of Western Australia (2021/ET000377) and the Office of the Director-General of Health Sciences from the Ministry of Health, Republic of Indonesia, through Hasanuddin University (LB.02.01/2.2/6807/2022).

### Visualisation and assessment

**Fig. 2:**
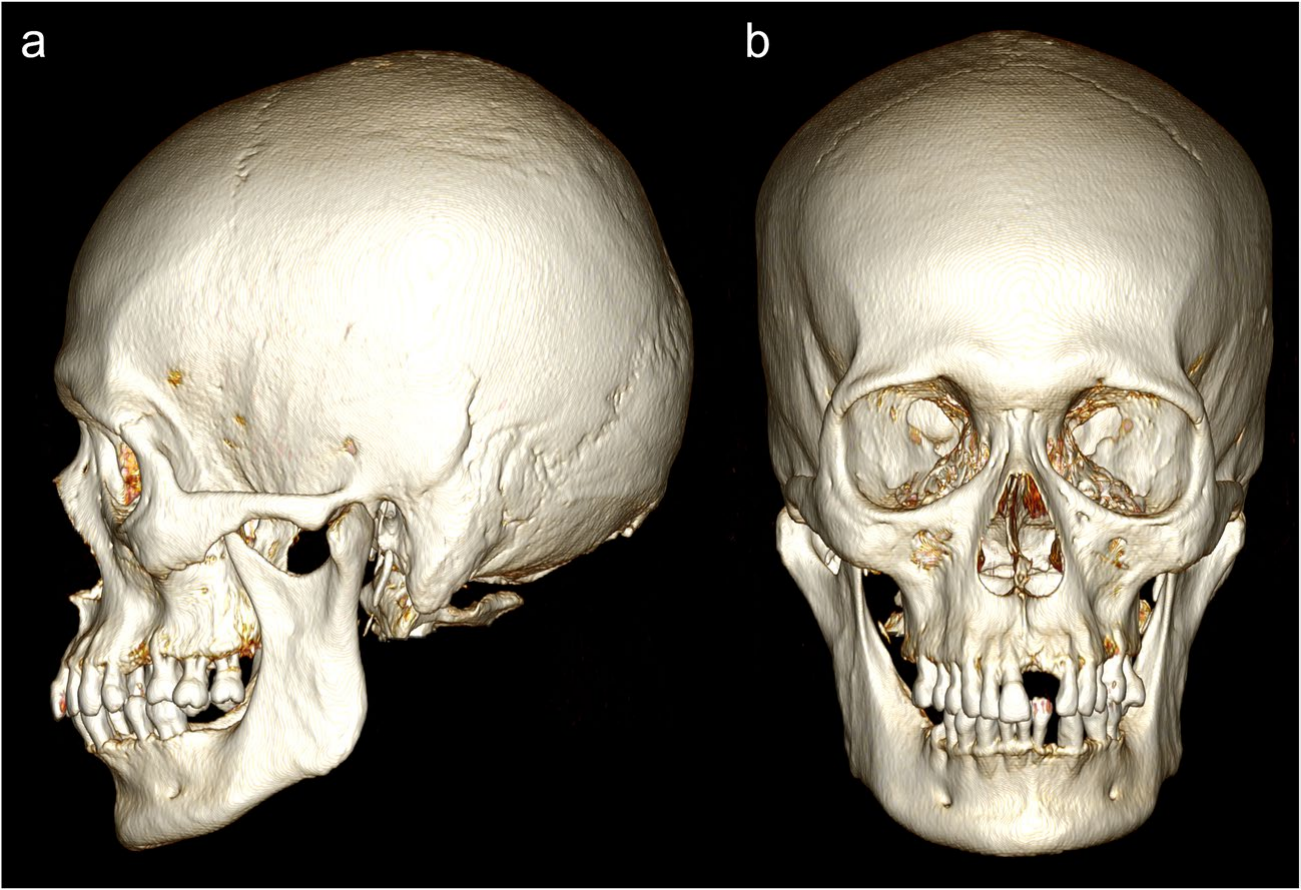
3D visualisation of the skull in (a) left lateral and (b) anterior view

**Fig. 3:**
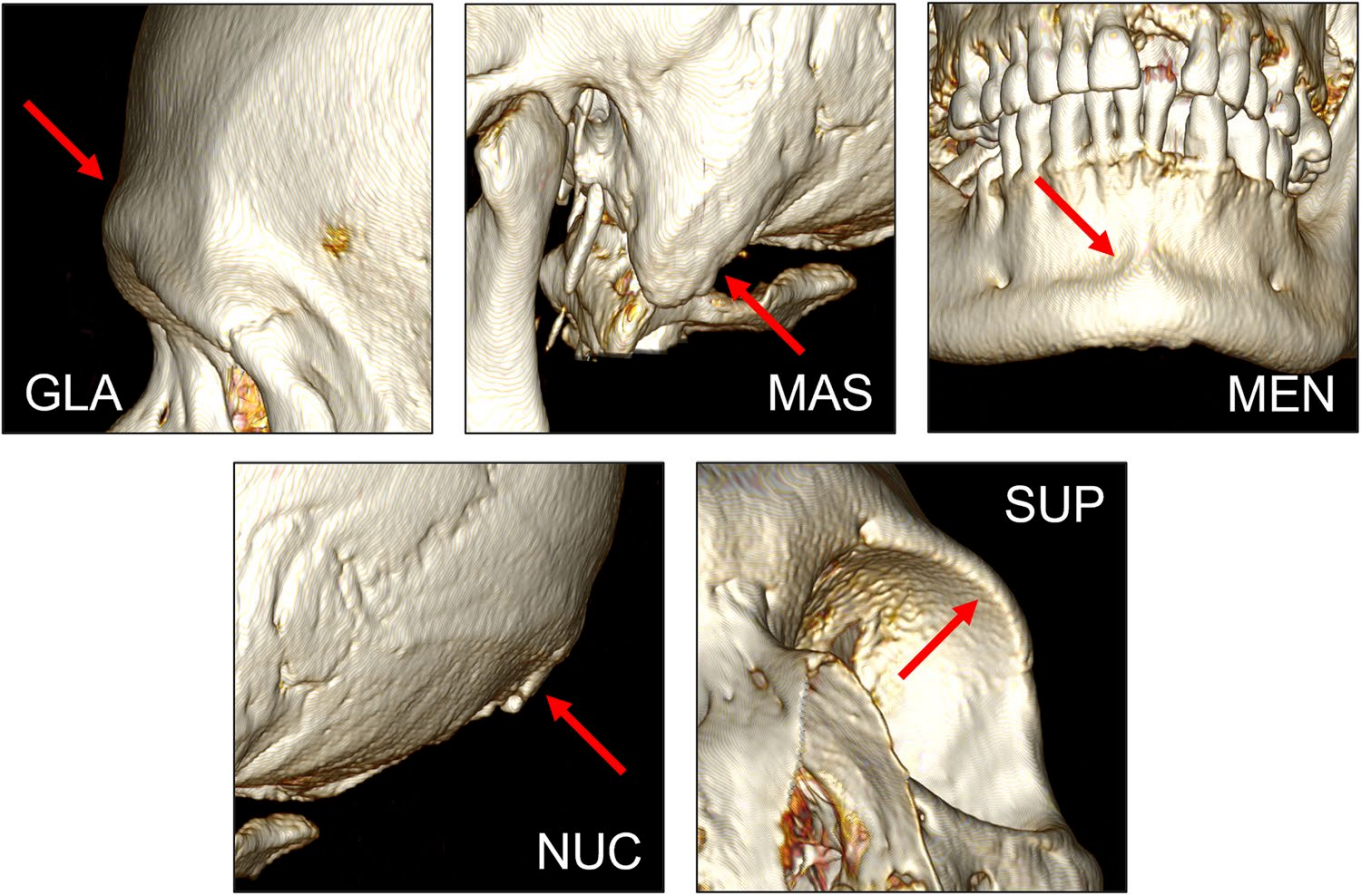
The five cranial traits used in the Walker [5] standard visualised in a 3D environment: glabella (GLA), mastoid process (MAS), mental eminence (MEN), nuchal crest (NUC), and supraorbital margin (SUP)

Visualisation was performed using the “3D volume rendering” function in *OsiriX® version 13.0.1*. To orientate each scan to its respective views for assessment, both the “3D rotate” and “pan” functions were used; an example 3D visualisation is shown in Fig. 2. The “High Contract” 3D preset was used in the volume rendering window. CLUT was set to “VR Muscles-Bones”, and no convolution filters were applied. Following Walker [5], scores were then assigned for each of the five traits, including any bilateral traits: glabella (GLA), mastoid process (MAS), mental eminence (MEN), nuchal crest (NUC), and supraorbital margin (SUP). The virtual representations of the five assessed features are depicted in Fig. 3.

Each cranial trait is scored individually on an ordinal five-point scale from 1 indicating “minimal expression” to 5 indicating “maximal expression.” To aid in scoring, visual illustrations and written descriptions were provided. No associations with masculinity or femininity were noted for any of the scores in the 2008 paper, although such associations did appear in *Standards* [6]: traits scored as 1 or 2 were assigned as female; a score of 3 was ambiguous; and scores 4 or 5 were male. The derivation of composite scores in *Standards* [6] assumed that all cranial traits were weighted equally. Given the latter assumption is unlikely biologically robust, traits were weighted against their individual discriminatory power in Walker [5].

### Statistical analysis

All statistical analyses were performed on *IBM SPSS Statistics version 29.0.0*.

#### Intra-observer agreement

A subset of 50 MSCT scans were used to quantify intra-observer agreement: 24 female (mean age = 44.3 years, SD = 13.6 years) and 26 male (mean age = 44.8 years, SD = 16.0 years). These scans were assessed by the lead author a total of three times; repeat assessments were performed with an interval of at least 24 h. For all bilateral traits, only the left side was scored. Agreement was calculated using the intraclass correlation (ICC) statistic; interpretation and its associated values are as followed: poor (ICC < 0.50), moderate (0.50 ≤ ICC ≤ 0.75), good (0.75 < ICC ≤ 0.90), and excellent (ICC > 0.90) [28].

#### Bilateral asymmetry

Previous studies utilising the Walker [5] standard did not include assessments of bilateral asymmetry (e.g., [15, 18]), implying that these authors followed scoring procedures from the original publication, or that the left side was used unless damaged or missing (e.g., [8, 16]). The present study included assessments of bilateral traits to compare the differences in scoring and its potential effects in estimating skeletal sex. Asymmetry was assessed using a Wilcoxon signed-rank test (*Z*).

#### Trait score distributions

Trait score frequency distributions are calculated and sorted by sex, with differences in sex-based distributions assessed using the Mann–Whitney U test. Sex classification accuracies based on trait frequency distributions are derived from the probability of obtaining a specific score for a single trait. This probability is calculated from the proportionality of scores assigned against the distribution. In this instance, the probability of an individual being classified as female is calculated using the following equation:

$$pf=\frac{\mathrm{\% }female}{\mathrm{\% }female+\mathrm{\% }male}$$

where % *female* and % *male* are the proportions of females and males assigned a specific score for the trait being assessed. The probability of an individual being classified as male would be calculated based on the following equation:

$$pm=1-pf$$

If *pf* > 0.50, the individual is likely female; if *pm* > 0.50, the individual is likely male.

#### Validation of Walker (2008) and Tallman (2019) functions

Nine multivariate functions from Walker [5] are applied to the Indonesian sample to assess the validity of this standard developed on European and Native American populations in the Indonesian sample. Moreover, fifteen functions incorporating the Japanese, Thai, and a pooled population group in Tallman [16] are applied to analyse the performance of Asian-derived predictive models and their suitability for forensic application in Indonesia. Differences in performance are assessed by comparing classification accuracies and sex bias values of each function. Sex bias values are calculated as the difference between the female and male classification accuracies; positive values overestimate females while negative values overestimate males.

#### Univariate and multivariate models for the indonesian population

Univariate predictive models only include one trait as the independent variable, while multivariate predictive models incorporate various trait combinations based the absence of one or more traits if the skull were fragmented (e.g., missing mandible, and therefore no mental eminence to score). To derive and test univariate and multivariate predictive models specific to an Indonesian population, 75% of the sample are randomly selected for training, with the remaining 25% used as a hold-out for validation. Sex is coded as 0 for females and 1 for males. Binary logistic regression (BLR) was utilised to derive these predictive models. Sex-specific and total classification accuracies, posterior probabilities, and sex bias values are calculated based on the trait combinations used; training and validation accuracies are provided.

## Results

### Intra-observer agreement

The ICC estimates and 95% confidence interval (CI) for intra-observer agreement on each trait was based on a single-rating, absolute agreement, 2-way mixed-effect model. The glabella had excellent agreement, ICC = 0.926, 95% CI [0.885, 0.954]. The nuchal crest, ICC = 0.863, 95% CI [0.792, 0.914], mastoid process, ICC = 0.798, 95% CI [0.701, 0.872], and supraorbital margin, ICC = 0.762, 95% CI [0.653, 0.848], had good agreement. The mental eminence had moderate agreement, ICC = 0.730, 9% CI [0.610, 0.825].

### Bilateral asymmetry

Wilcoxon signed-rank tests reported no significant differences in the assignment of scores for the mastoid process, *Z* = –1.26, *p* = 0.208, and supraorbital margin, *Z* = –1.40, *p* = 0.161. Consequently, scores recorded from the left side only were used for all subsequent statistical analyses of these traits.

### Trait score distributions

Sex-based variations in trait score distribution frequencies were significantly different for all traits: glabella, *U* = 10.17, *p* < 0.001; mastoid process, *U* = 8.02, *p* < 0.001; mental eminence, *U* = 5.81, *p* < 0.001; nuchal crest, *U* = 5.83, *p* < 0.001; supraorbital margin, *U* = 3.99, *p* < 0.001. Cranial trait score distributions for the Indonesian population are provided in Table 1. All score expressions were recorded for each trait, except for the mental eminence, for which no female had a recorded score of 5. In general, lower trait scores are associated with females, while higher traits scores are associated with males. Males were also observed to have a wider range in cranial trait expression compared to females.

**Table 1:** Frequency distribution of cranial trait scores and their proportions sorted by sex

	Score Frequency				
	1	2	3	4	5
Cranial Trait^a^	*n* (%)	*n* (%)	*n* (%)	*n* (%)	*n* (%)
GLA					
Female	49 (56.3)	29 (33.3)	6 (6.9)	2 (2.3)	1 (1.1)
Male	9 (7.8)	13 (11.2)	19 (16.4)	36 (31.0)	39 (33.6)
MAS					
Female	9 (10.3)	64 (73.6)	8 (9.2)	4 (4.6)	2 (2.3)
Male	2 (1.8)	25 (22.1)	39 (34.5)	30 (26.5)	17 (15.0)
MEN					
Female	32 (36.8)	24 (27.6)	20 (23.0)	11 (12.6)	0 (0.0)
Male	10 (8.8)	20 (17.7)	44 (38.9)	24 (21.2)	15 (13.3)
NUC					
Female	42 (48.3)	16 (18.4)	16 (18.4)	8 (9.2)	5 (5.7)
Male	12 (10.6)	22 (19.5)	32 (28.3)	33 (29.2)	14 (12.4)
SUP					
Female	11 (12.6)	19 (21.8)	29 (33.3)	13 (14.9)	15 (17.2)
Male	5 (4.4)	7 (6.2)	33 (29.2)	40 (35.4)	28 (24.8)

^a^ GLA = glabella; MAS = mastoid process; MEN = mental eminence; NUC = nuchal crest; SUP = supraorbital margin

Probability values associated with each character state per trait are presented in Table 2. Based on these probability values, the glabella, mastoid process, and mental eminence had scores of 1 and 2 represent female individuals, while scores from 3 to 5 represented male individuals. By comparison, nuchal crest scores of 1 represented female individuals, and 2 to 5 represented male individuals. In contrast, supraorbital margin scores from 1 to 3 represented female individuals, and scores from 4 to 5 represented male individuals.

**Table 2:** Probabilities associated with sex classification based on assigned trait score. See Table 1 for trait score frequency distributions

	Score Probabilities				
Cranial Trait^a^	1	2	3	4	5
GLA					
Female	.879	.748	.296	.069	.033
Male	.121	.252	.704	.931	.967
MAS					
Female	.854	.769	.210	.148	.133
Male	.146	.231	.790	.852	.867
MEN					
Female	.806	.609	.371	.373	0
Male	.194	.391	.629	.627	1
NUC					
Female	.820	.486	.394	.239	.317
Male	.180	.514	.606	.761	.683
SUP					
Female	.741	.779	.533	.297	.410
Male	.259	.221	.467	.703	.590

^a^ GLA = glabella; MAS = mastoid process; MEN = mental eminence; NUC = nuchal crest; SUP = supraorbital margin

Table 3 summarises differences in cranial trait score distributions between the Indonesian population and the five population groups in Walker [5] and Tallman [16]. When compared against the American/English and Native American populations in Walker [5], the mental eminence was noted to be more gracile than the Native American population group, while the nuchal crest was more gracile than both the English/American and Native American population groups. In contrast, the supraorbital margin was more robust in the Indonesian population compared to the two population groups in Walker [5].

**Table 3:** Differences in cranial trait score distributions based on probability of sex classification sorted by trait, with population groups from Walker [5] and Tallman [16]

		Score Threshold for Sex Classification				
Trait^a^	Population Group	1	2	3	4	5
GLA	Indonesian	F		M		
	Thai	F	M			
	Filipino	F	M			
	Japanese	F	M			
	American/English	F		M		
	Native American	F		M		
MAS	Indonesian	F		M		
	Thai	F			M	
	Filipino	F			M	
	Japanese	F		Ind.^b^	M	
	American/English	F		M		
	Native American	F		M		
MEN	Indonesian	F		M		
	Thai	F		M		
	Filipino	F		M		
	Japanese	F		M		
	American/English	F		M		
	Native American	F			M	
NUC	Indonesian	F	M			
	Thai	F		M		
	Filipino	F		M		
	Japanese	F			M	
	American/English	F		M		
	Native American	F		M		
SUP	Indonesian	F			M	
	Thai	F		M		
	Filipino	F		M		
	Japanese	F		M		
	American/English	F	M			
	Native American	F		M		

^a^ GLA = glabella; MAS = mastoid process; MEN = mental eminence; NUC = nuchal crest; SUP = supraorbital margin

^b^ Japanese individuals who scored 3 on the mastoid process were considered indeterminate

### Validation of Walker (2008) and Tallman (2019) functions

The nine functions (W1 to W9) provided in Walker [5] were applied to the Indonesian population as reported in Table 4. Performance varied depending on the function used. The most accurate function was W1, originally developed for an American/English population, with classification accuracies of 87.4% in Indonesian females and 88.5% in Indonesian males, compared to 86.4% and 88.4% as reported by Walker [5], respectively. Function W5, also developed for an American/English population, had the lowest classification accuracy for Indonesian females at 44.8% and the highest classification accuracy for Indonesian males at 92.0%. The sex bias values in the Indonesian population ranged from –47.2% in Function W5 to 27.4% in Function W8, while Walker [5] reported sex bias values from –4.5% to 13.4%. All American/English functions (W1 to W6) misclassified females (i.e., negative sex bias). In contrast, all Native American functions (W7 to W9) misclassified males (i.e., positive sex bias).

**Table 4:** Classification accuracies and sex biases of the nine Walker [5] functions, applied to the Indonesian population

			Accuracy (%)			Sex Bias (%)	
Population Group/Function^a^		Sex	This Study	Walker	Diff.^b^	This Study	Walker
American/English							
W1	1.375(GLA) + 1.185(MAS) + 1.151(MEN) – 9.128	Female	87.4	86.4	1.0	–1.1	–2.0
		Male	88.5	88.4	0.1		
W2	1.568(GLA) + 1.459(MAS) – 7.434	Female	81.6	82.9	–1.3	–8.7	–2.5
		Male	90.3	85.4	4.9		
W3	1.525(GLA) + 1.485(MEN) – 7.372	Female	75.9	82.1	–6.2	–12.6	–4.5
		Male	88.5	86.6	1.9		
W4	1.629(MEN) + 1.415(MAS) – 7.382	Female	63.2	83.6	–20.4	–20.9	3.7
		Male	84.1	79.9	4.2		
W5	1.007(SUP) + 1.850(MEN) – 6.018	Female	44.8	77.9	–33.1	–47.2	–0.2
		Male	92.0	78.1	13.9		
W6	0.7(NUC) + 1.559(MAS) – 5.329	Female	74.7	82.9	–8.2	–7.6	6.1
		Male	82.3	76.8	5.5		
Native American							
W7	0.499(SUP) + 0.606(MEN) – 3.414	Female	75.9	77.9	–2.0	21.0	–0.2
		Male	54.9	78.1	–23.2		
W8	0.576(MEN) + 1.136(MAS) – 4.765	Female	92.0	72.7	19.1	27.4	–1.4
		Male	64.6	74.1	–9.5		
W9	0.797(GLA) + 1.085(MAS) – 5.025	Female	88.5	82.9	5.6	4.4	13.4
		Male	84.1	69.5	14.6		

^a^ GLA = glabella; MAS = mastoid process; MEN = mental eminence; NUC = nuchal crest; SUP = supraorbital margin

^b^ Differences in classification accuracy between this study and published values in Walker [5]

Seventeen functions (Japanese: J1 to J5; Thai: T1 to T5; and pooled Japanese/Thai: P1 to P7) in Tallman [16] were also applied to the Indonesian population and their results are detailed in Table 5. The most accurate function was T2, with classification accuracies of 72.4% in Indonesian females and 93.8% in Indonesian males, compared to 76.3% and 89.6% in Tallman [16], respectively. The least accurate functions were T1 and P7, with classification accuracies of 50.6% in Indonesian females and 99.1% in Indonesian males, compared to 86.7% and 84.1% in Function T1, and 72.7% and 91.7% in Function P7, respectively. All functions had overly large negative sex bias values that were greater than those reported in the original study.

**Table 5:** Classification accuracies and sex biases of the seventeen Tallman [16] functions, applied to the Indonesian population

			Accuracy (%)			Sex Bias (%)	
Population Group/Function^a^		Sex	This Study	Tallman	Diff.^b^	This Study	Tallman
Japanese							
J1	0.966(MAS) + 1.917(GLA) – 4.990	Female	55.2	82.3	–27.1	–41.3	–5.2
		Male	96.5	87.5	9.0		
J2	2.290(GLA) – 2.417	Female	56.3	89.7	–33.4	–38.4	17.4
		Male	94.7	72.3	22.4		
J3	0.968(MAS) + 1.851(GLA) + 0.743(MEN) – 6.618	Female	58.6	76.2	–17.6	–40.5	–13.4
		Male	99.1	89.6	9.5		
J4	0.356(NUC) + 0.989(MAS) + 0.516(SUP)	Female	54.0	73.0	–19.0	–46.0	–16.5
	+ 1.707(GLA) + 0.799(MEN) – 8.194	Male	100.0	89.5	10.5		
J5	0.588(NUC) + 0.904(MAS) + 1.948(GLA)	Female	66.7	73.7	–7.0	–29.8	–13.7
	+ 0.886(MEN) – 8.537	Male	96.5	87.4	9.1		
Thai							
T1	1.227(MAS) + 2.147(GLA) – 6.495	Female	50.6	86.7	–36.1	–48.5	2.6
		Male	99.1	84.1	15.0		
T2	1.157(MAS) + 1.657(GLA) + 0.707(MEN) – 7.376	Female	72.4	76.3	–3.9	–21.4	–13.3
		Male	93.8	89.6	4.2		
T3	0.356(NUC) + 0.989(MAS) + 0.516(SUP)	Female	69.0	74.3	–5.3	–30.1	–17.2
	+ 1.471(GLA) + 0.617(MEN) – 8.528	Male	99.1	91.5	7.6		
T4	0.990(MAS) + 0.472(SUP) + 2.024(GLA)	Female	65.5	85.4	–19.9	–34.5	–7.0
	+ 0.740(MEN) – 8.559	Male	100.0	92.4	7.6		
T5	2.829(GLA) – 3.161	Female	56.3	87.8	–31.5	–38.4	8.4
		Male	94.7	79.4	15.3		
Pooled Japanese/Thai							
P1	1.012(MAS) + 2.227(GLA) – 5.691	Female	55.2	82.4	–27.2	–41.3	2.2
		Male	96.5	80.2	16.3		
P2	0.873(MAS) + 0.478(SUP) + 1.585(GLA)	Female	57.5	79.5	–22.0	–42.5	–11.8
	+ 0.496(MEN) – 6.816	Male	100.0	91.3	8.7		
P3	2.458(GLA) – 2.732	Female	56.3	89.2	–32.9	–38.4	17.7
		Male	94.7	71.5	23.2		
P4	0.491(NUC) + 0.832(MAS) + 0.590(SUP)	Female	64.4	68.4	–4.0	–34.7	–26.2
	+ 1.543(GLA) + 0.689(MEN) – 8.628	Male	99.1	94.6	4.5		
P5	0.554(NUC) + 0.855(MAS) + 1.963(GLA)	Female	66.7	76.8	–10.1	–29.8	–10.7
	+ 0.747(MEN) – 8.098	Male	96.5	87.5	9.0		
P6	1.067(MAS) + 2.106(GLA) + 0.530(MEN)	Female	65.5	81.7	–16.2	–31.8	–0.5
	– 7.055	Male	97.3	82.2	15.1		
P7	1.018(MAS) + 0.616(SUP) + 1.657(GLA)	Female	50.6	72.7	–22.1	–48.5	–19.0
	– 6.481	Male	99.1	91.7	7.4		

^a^ GLA = glabella; MAS = mastoid process; MEN = mental eminence; NUC = nuchal crest; SUP = supraorbital margin

^b^ Differences in classification accuracy between this study and published values in Tallman [16]

**Table 6:** Indonesia-specific functions and their associated training and validation classification accuracies, along with sex bias

		Training Accuracy (%)				Validation Accuracy (%)			
Indonesia-Specific Predictive Models^a^		Female	Male	Total	Bias (%)	Female	Male	Total	Bias (%)
Univariate									
S1	1.666(GLA) – 4.123	89.4	85.7	87.3	3.7	90.5	75.9	82.0	14.6
S2	1.564(MAS) – 3.934	84.9	81.0	82.7	3.9	81.0	62.1	80.0	18.9
S3	0.743(MEN) – 1.708	62.1	71.4	67.3	–9.3	71.4	79.3	76.0	–7.9
S4	0.815(NUC) – 1.872	66.7	75.0	71.3	–8.3	66.7	55.2	60.0	11.5
S5	0.663(SUP) – 2.002	71.2	66.7	68.7	4.5	57.2	41.4	48.0	15.8
Multivariate									
M1	1.615(GLA) + 0.330(SUP) – 5.102	89.4	85.7	87.3	3.7	90.5	75.9	82.0	14.6
M2	1.753(MAS) + 0.950(SUP) – 7.725	77.3	85.7	82.0	–8.4	61.9	65.5	64.0	–3.6
M3	1.501(GLA) + 1.073(MAS) – 6.604	86.4	89.3	88.0	–2.9	90.5	82.8	86.0	7.7
M4	1.391(GLA) + 1.431(MAS) + 0.744(SUP) – 9.789	86.4	89.3	88.0	–2.9	90.5	82.8	86.0	7.7
M5	1.588(MAS) + 0.631(NUC) + 0.837(SUP) – 8.601	78.8	88.1	84.0	–9.3	61.9	65.5	64.0	–3.6
M6	1.527(GLA) + 1.011(MAS) + 0.610(MEN) – 7.991	89.4	88.1	88.7	1.3	95.2	82.8	88.0	12.5
M7	1.305(GLA) + 1.298(MAS) + 0.384(NUC)	87.9	86.9	87.3	1.0	90.5	79.3	84.0	11.2
	+ 0.632(SUP) – 9.867								
M8	1.434(GLA) + 1.376(MAS) + 0.559(MEN)	84.9	88.1	86.7	–3.3	85.7	82.8	84.0	2.9
	+ 0.707(SUP) – 10.999								
M9	1.350(GLA) + 1.202(MAS) + 0.598(MEN)	89.4	89.3	89.3	0.1	95.2	82.8	88.0	12.5
	+ 0.433(NUC) + 0.581(SUP) – 11.134								

^a^ GLA = glabella; MAS = mastoid process; MEN = mental eminence; NUC = nuchal crest; SUP = supraorbital margin

### Univariate Indonesian-specific predictive models

Five univariate predictive models (S1 to S5) derived for each cranial trait are detailed in Table 6. Function S1 (glabella) had the highest classification accuracy in the training sample, with 89.4% for females and 85.7% for males. Function S3 (mental eminence) had the lowest classification accuracy in the training sample at 62.1% for females. In the validation (i.e., hold-out) sample, Function S1 had the highest classification accuracy of 90.5% for females and 75.9% for males, while Function S5 (supraorbital margin) had the lowest accuracy of 57.2% for females and 41.4% for males. The sex bias values in the training sample ranged from –9.3% in Function S3 (mental eminence) to 3.7% in Function S1 (glabella). For the validation sample, the sex bias was lowest at –7.9% for Function S3 (supraorbital margin) and largest at 18.9% for Function S2 (mastoid process).

### Multivariate Indonesian-specific predictive models

Nine multivariate predictive models (M1 to M9) are also detailed in Table 6. Function M9, which incorporated all traits, had the highest training classification accuracy at 89.4% for females and 89.3% for males. Function M2 (mastoid process and supraorbital margin) had the lowest training classification accuracy at 77.3% for females and 85.7% for males. In the validation sample, Functions M6 (glabella, mastoid process, and mental eminence) and M9 had the highest classification accuracies at 95.2% for females and 82.8% for males. Functions M2 and M5 (mastoid process, nuchal crest, and supraorbital margin) had the lowest classification accuracies at 61.9% for females and 65.5% for males. The best performing function overall (i.e., high classification accuracy and low sex bias) was M8, which included all traits except the nuchal crest, had classification accuracies of 85.7% for females and 82.8% for males. Sex biases values for the training sample ranged from –9.3% in Function M5 to 3.7% in Function M1. For the validation sample, sex bias was smallest at –3.6% in Functions M2 and M5 and largest at 14.6% in Function M1.

## Discussion

This aim of the present study was to evaluate the effectiveness of the Walker [5] and Tallman [16] BLR functions in a contemporary Indonesian population. Based on the results presented, classification accuracies were lower than those of the original publications and demonstrated the need for population-specific models to ensure high classification accuracies are maintained. Fourteen functions derived from the Indonesian population provide classification accuracies that are comparable to those found in Walker [5] and Tallman [16]. These functions would therefore serve to improve the capabilities of forensic practitioners in Indonesia for both routine medicolegal casework and DVI.

### Observer agreement

Intra-observer agreement for all five traits was moderate to excellent, with similar results reported in other studies [5, 9, 16]. As with any morphoscopic standard, visual illustrations and written descriptions should be clear and concise to minimise ambiguity in trait scoring [29]. Some descriptors in Walker [5] involve tactile interactions with the feature (i.e., mental eminence and supraorbital margin). Inter-observer agreement was not considered for this study as the Walker [5] standard is amongst the most widely used by forensic practitioners [4]. There is thus considerable published data showing acceptable levels of inter-observer agreement when applied in both physical [9, 13–15] and digital modalities [17].

With the use of digital 3D volume-rendered CT scans, this tactile approach is not feasible and would require modifying descriptors to more appropriately reflect the visual observations made. Dereli et al. [17] likewise noted the lack of tactile interactions and suggested incorporating other identifying features as an alternative. To address these issues, instructions for the examination of cranial traits in a digital 3D environment were provided in the software package *MorphoPASSE* developed by Klales [30] to aid observers in scoring.

The mental eminence appears to be a contentious trait in skeletal sex estimation due to its low observer agreement. Several studies report the mental eminence having the lowest observer agreement [9, 31]. As such, these authors have suggested the mental eminence not be considered. However, other studies have demonstrated more favourable observer agreements for the mental eminence (e.g., [16–18]), including the present study. In relation to the latter, the orientation of the mental eminence within the 3D environment in lieu of tactile feedback allowed for more consistent scoring (see Fig. 3).

### Variation in trait score distribution between population groups

As detailed in Table 3, when assessing the trait score distributions from the Indonesian population against the Japanese, Filipino, and Thai population groups in Tallman [16], the glabella and supraorbital margin were more robust in the Indonesian population, while the mastoid process and nuchal crest were more gracile. Similar trait score distributions were observed only for the mental eminence. These observed variations support other studies that highlight the importance of developing and utilising population-specific standards, since different population groups display varying degrees of sex-based differences even if they originate from the same continent [8, 16].

Applying the nine functions of Walker [5] to the Indonesian sample expectedly resulted in different classification accuracies to the original, with differences ranging from –33.1% to 5.6% in females and from –23.2% to 14.6% in males, depending on the function used. All American/English functions had larger male sex bias values, while the Native American functions had larger female sex bias values. Similarly, testing the Japanese, Thai, and pooled-population functions by Tallman [16] resulted in classification accuracies with similarly large differences from –36.1% to –3.9% for females and from 4.2% to 23.2% in males. All functions had larger female sex biases, regardless of the population group being tested, including the pooled population.

It is evident on the basis of cranial trait distributions in the Indonesian population compared to the Filipino, Japanese, and Thai populations, that there are marked distribution differences in four of the five traits. These results show the variation in cranial trait expression across population groups, as suggested by other similar studies [9, 15, 16]. Such variations can be attributed to a variety of intrinsic factors such as diet and socioeconomic status [32], and extrinsic factors, including geographic locale and the environment [33].

### Indonesian-specific predictive models

A total of 14 predictive models, five univariate and nine multivariate, were derived based on the Indonesian training sample. For univariate functions, the glabella performed best in the training subset, with classification accuracies at 89.4% for females, 85.7% for males, and a sex bias of 3.7%. It was also the best performing in the validation subset, with classification accuracies at 90.5% for females and 75.9% for males. However, its 14.6% sex bias is not within acceptable limits (i.e., absolute difference greater than 5.0%). All univariate models in this study had total classification accuracies below 85.0% and sex biases outside of acceptable limits. Therefore, use of these models in forensic practice is not recommended.

Multivariate functions had training classification accuracies from 77.3% to 89.4% for females, 85.7% to 89.3% for males, and sex biases from –9.3% to 3.7%, depending on the combination of traits used. Likewise, validation classification accuracies ranged from 61.9% to 95.2% for females, 65.5% to 82.8% for males, and sex biases from –3.6% to 14.6%. The best performing trait combination on the training subset, Function M9, included all traits as variables, with classification accuracies at 89.4% for females, 89.3% for males, and a sex bias of 0.1%. The validation subset, Function M8, included all traits except the nuchal crest as variables, with classification accuracies at 85.7% for females, 82.8% for males, and a sex bias of 2.9%. The sex bias for both these functions are within acceptable limits.

The selection of multivariate functions is dependent on the availability of traits for scoring. As the likelihood of recovering fragmentary cranial elements is high, the trait combinations presented in this study are region-specific. For example, Function M7 would be appropriate if only the mandible were missing, and therefore the mental eminence cannot be scored. Likewise, Function M1 would be appropriate when only the frontal bone is recovered, and as such the glabella and supraorbital margin may be scored.

**Fig. 4:**
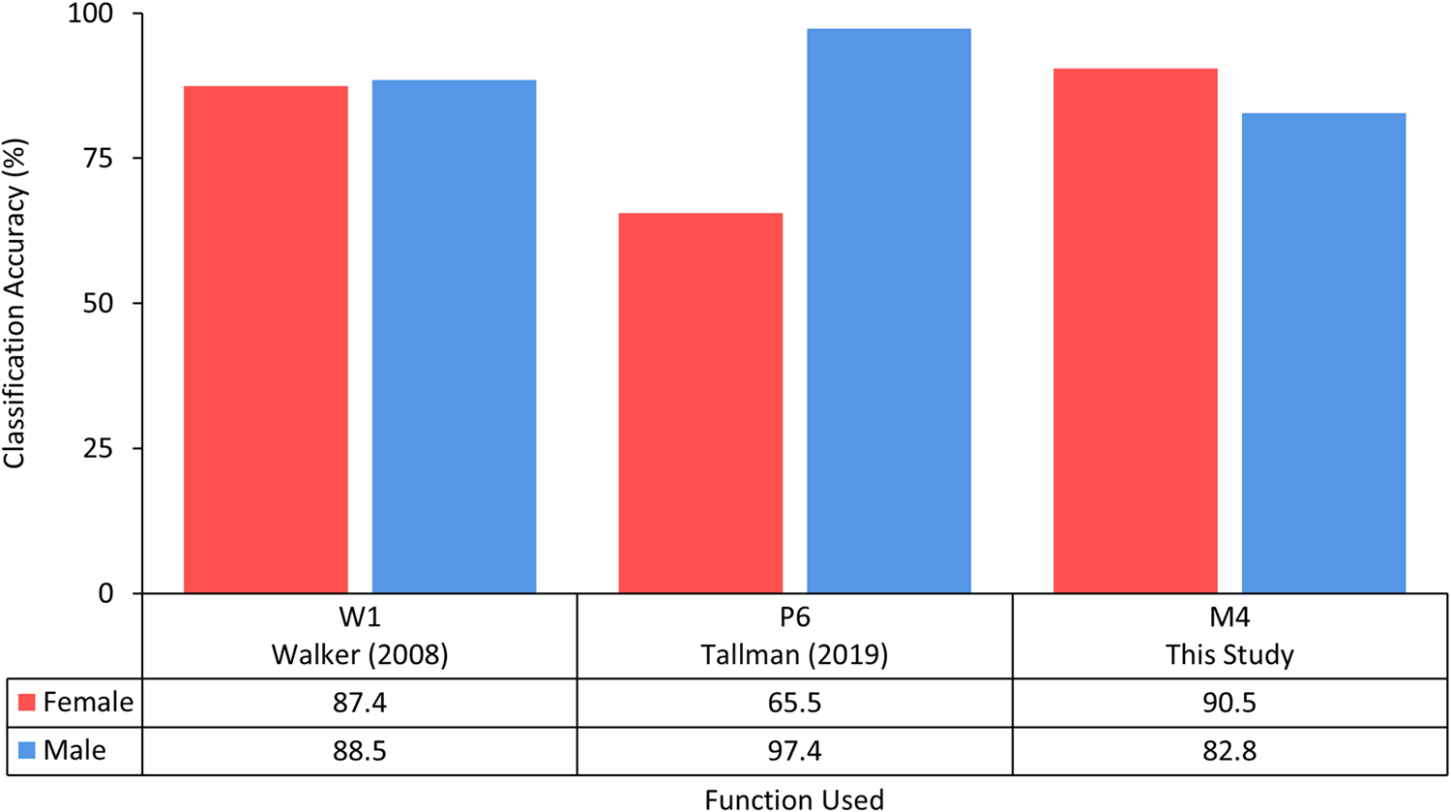
Sex-specific classification accuracies derived from functions with predictors glabella, mastoid process, and mental eminence presented in Walker [5], Tallman [16], and this study when applied to the present Indonesian sample

Figure 4 visualises the differences in classification accuracies when Function W1 in Walker [5] and P6 in Tallman [16] are applied to the Indonesian sample, and M4 being derived from the study sample. All three functions include the same traits as variables: the glabella, mastoid process, and mental eminence. It demonstrated the improvements in classification accuracy when population-specific functions are applied, which further supports the need for predictive models developed for the population group being assessed.

### Study limitations

As Indonesia is an island archipelago with a long and rich history, it is important to acknowledge the movement of different subpopulations both into and within the country [34, 35]. The application of the presented models to the broader Indonesian population must be tested. As is the case in the evaluation of cranial sex estimation standards in other large and geographically disparate populations in countries like Australia [36, 37], it is likely that adjustments to these models will be needed when larger samples from different regions of Indonesia become available for analysis in the future.

## Conclusion

This study has validated the use of the Walker [5] standard for sex estimation in a sample of Indonesian cranial CT scans. By testing the application of BLR functions from the original Walker [5] standard and from other Asian-derived populations outside of Indonesia, this study demonstrated their limitations when applied to the Indonesian sample. Consequently, the development of Indonesian-specific models improved classification accuracies for use in modern forensic practice. Although there are regional studies that have focused on the utility of CT scans for metric data (e.g., [36]), the inclusion of morphoscopic standards into Indonesia’s forensic anthropological literature further expands the toolkit available to forensic practitioners in that jurisdiction. This will improve their capabilities, providing access to a readily deployable standard for skeletal sex estimation that does not require expensive specialised equipment.

## References

[1] Christensen AM, Passalacqua NV, Bartelink EJ (2019) Sex Estimation. Forensic Anthropology: Current Methods and Practice. 2nd edn. Academic Press, London, UK. 243–270. 10.1016/B978-0-12-815734-3.00008-7

[2] Rogers TL (2005). Determining the Sex of Human Remains Through Cranial Morphology. J Forensic Sci.

[3] Winburn AP, Yim A-D, Stock MK (2022). Recentering Forensic Anthropology Within a Multifaceted Body of Evolutionary Theory: Strengthening Method by Making Theory Explicit. Am J Biol Anthropol.

[4] Klales AR (2020) Practitioner Preference for Sex Estimation from Human Skeletal Remains. In: Klales AR, ed. Sex Estimation of the Human Skeleton: History, Methods, and Emerging Techniques. Academic Press, London, UK. 11–23. 10.1016/B978-0-12-815767-1.00002-X

[5] Walker PL (2008). Sexing Skulls Using Discriminant Function Analysis of Visually Assessed Traits. Am J Phys Anthropol.

[6] Buikstra JE, Ubelaker DH (1994) Standards for Data Collection from Human Skeletal Remains. Proceedings of a Seminar at The Field Museum of Natural History. Arkansas Archaeological Survey, Fayetteville, AR.

[7] Avent PR, Hughes CE, Garvin HM (2022). Applying Posterior Probability Informed Thresholds to Traditional Cranial Trait Sex Estimation Eethods. J Forensic Sci.

[8] Garvin HM, Sholts SB, Mosca LA (2014). Sexual Dimorphism in Human Cranial Trait Scores: Effects of Population, Age, and Body Size. Am J Phys Anthropol.

[9] Lewis CJ, Garvin HM (2016). Reliability of the Walker Cranial Nonmetric Method and Implications for Sex Estimation. J Forensic Sci.

[10] Grivas CR, Komar DA (2008). Kumho, Daubert, and the Nature of Scientific Inquiry: Implications for Forensic Anthropology. J Forensic Sci.

[11] National Research Council (2009). Strengthening Forensic Science in the United States: A Path Forward.

[12] Franklin D (2023) Estimation of Skeletal Sex. In: Houck MM, ed. Encyclopedia of Forensic Sciences. 3rd edn. Elsevier, Oxford. 292–303. 10.1016/B978-0-12-823677-2.00098-2

[13] Oikonomopoulou E-K, Valakos E, Nikita E (2017). Population-Specificity of Sexual Dimorphism in Cranial and Pelvic Traits: Evaluation of Existing and Proposal of New Functions for Sex Assessment in a Greek Assemblage. Int J Legal Med.

[14] Cappella A, Bertoglio B, Di Maso M (2022). Sexual Dimorphism of Cranial Morphological Traits in an Italian Sample: A Population-Specific Logistic Regression Model for Predicting Sex. Biology.

[15] Krüger GC, L’Abbé EN, Stull KE, Kenyhercz MW (2015). Sexual Dimorphism in Cranial Morphology Among Modern South Africans. Int J Legal Med.

[16] Tallman SD (2019). Cranial Nonmetric Sexual Dimorphism and Sex Estimation in East and Southeast Asian Individuals. Forensic Anthropol.

[17] Dereli AK, Zeybek V, Sagtas E, Senol H, Ozgul HA, Acar K (2018). Sex Determination with Morphological Characteristics of the Skull by Using 3D Modeling Techniques in Computerized Tomography. Forensic Sci Med Pathol.

[18] Kelley SR, Tallman SD (2022). Population-Inclusive Assigned-Sex-at-Birth Estimation from Skull Computed Tomography Scans. Forensic Sci.

[19] Klales AR (2021). Current State of Sex Estimation in Forensic Anthropology. Forensic Anthropol.

[20] Traithepchanapai P, Mahakkanukrauh P, Kranioti EF (2016). History, Research and Practice of Forensic Anthropology in Thailand. Forensic Sci Int.

[21] Go MC (2018). Appraising Forensic Anthropology in the Philippines: Current Status and Future Directions. Forensic Sci Int.

[22] Go MC, Tallman SD, Kim J (2019). Advances in Forensic Anthropological Research in East and Southeast Asia. Forensic Anthropol.

[23] Avsar A, Okdemir E, Keten A, Kaya Ö (2019). Religion, Culture, and Autopsy: A Survey with Muslim Religious Officials. Am J Forensic Med Pathol.

[24] Sajid MI (2016). Autopsy in Islam: Considerations for Deceased Muslims and Their Families Currently and in the Future. Am J Forensic Med Pathol.

[25] Black S (2016) Disaster Anthropology: The 2004 Asian Tsunami. In: Blau S, Ubelaker DH, eds. Handbook of Forensic Anthropology and Archaeology. 2nd edn. Routledge, New York, NY. 507–519. 10.4324/9781315528939

[26] Indriati E (2014). Forensic Anthropological Roles in Disaster Victim Identification of Two Jakarta Hotels Bomb Blasts. Damianus J Med.

[27] Franklin D, Swift L, Flavel A (2016). 'Virtual Anthropology' and Radiographic Imaging in the Forensic Medical Sciences. Egypt J Forensic Sci.

[28] Koo TK, Li MY (2016). A Guideline of Selecting and Reporting Intraclass Correlation Coefficients for Reliability Research. J Chiropr Med.

[29] Walrath DE, Turner P, Brůžek J (2004). Reliability Test of the Visual Assessment of Cranial Traits for Sex Determination. Am J Phys Anthropol.

[30] Klales AR (2020) MorphoPASSE: Morphological Pelvis and Skull Sex Estimation Program. In: Klales AR, ed. Sex Estimation of the Human Skeleton: History, Methods, and Emerging Techniques. Academic Press, London, UK. 271–278. 10.1016/B978-0-12-815767-1.00016-X

[31] Williams BA, Rogers TL (2006). Evaluating the Accuracy and Precision of Cranial Morphological Traits for Sex Determination. J Forensic Sci.

[32] Franklin D, Flavel A (2019). Population Specificity in the Estimation of Skeletal Age and Sex: Case Studies Using a Western Australian Population. Aust J Forensic Sci.

[33] Stinson S (2012) Growth Variation: Biological and Cultural Factors. In: Stinson S, Bogin B, O'Rourke D, eds. Human Biology: An Evolutionary and Biocultural Perspective. 2nd edn. John Wiley & Sons, Inc, Hoboken, NJ. 587–635. 10.1002/9781118108062.ch12

[34] Tirtosudarmo R (2018) East Sulawesi Province: The Politics of Transcending Boundries. The Politics of Migration in Indonesia and Beyond. Springer, Singapore. 81–100. 10.1007/978-981-10-9032-5

[35] Husa K, Wohlschlägl H, Husa K, Trupp A, Wohlschlägl H (2014). Global Markets – Local Consequences: The Migration of Labour in Southeast Asia Since the Mid-Nineteenth Century. Southeast Asian Mobility Transitions: Issues and Trends in Migration and Tourism.

[36] Swift L, Obertova Z, Flavel A, Murray K, Franklin D (2022). Estimation of Sex from Cranial Measurements in an Australian Population. Aust J Forensic Sci.

[37] Swift L, Obertova Z, Franklin D (2023). Demonstrating the Empirical Effect of Population Specificity of Anthropological Standards in a Contemporary Australian Population. Int J Legal Med.

